# A QUALITATIVE STUDY IN COGNITIVE CARE PLANNING: THE ROLE OF FAMILY CAREGIVERS

**DOI:** 10.1093/geroni/igae098.0763

**Published:** 2024-12-31

**Authors:** Katherine Britt, Xaviera Xaio, Kevin Sun, Shaoqing Ge, Bin Huang

**Affiliations:** University of Iowa, Iowa City, Iowa, United States; BrainCheck, Inc., Austin, Texas, United States; BrainCheck, Inc., austin, Texas, United States; The University of Texas Austin, Austin, Texas, United States; BrainCheck, Austin, Texas, United States

## Abstract

Patients with cognitive impairment and dementia may benefit from cognitive care planning, a process of routinely and systematically evaluating patient needs and making lifestyle recommendations (i.e., physical exercise, healthier eating, medication recommendations, and advance care planning). Though dementia is progressive, tailored lifestyle recommendations and planning can improve emotional health, quality of life, and resource utilization to alleviate the disease burden. However, cognitive care planning is underutilized, and barriers to optimizing the process remain. We conducted a qualitative descriptive study with family care providers (N=9) who provide care for older adults, including patients with cognitive impairment and dementia, to explore the role of family caregivers in cognitive care planning with patients. Conventional content analysis was used. Several facilitators and barriers around family caregivers in cognitive care planning implementation were identified across four categories: uncertainty, burden, absence, and priority. Family caregivers were often older, experiencing their own health challenges, and uncertain how to get help and navigate the healthcare system. Family and caregivers’ presence during cognitive care planning was reported as a critical facilitator as it’s essential for gathering patient context, documenting changes in daily abilities, and facilitating provider communication. Our study has important implications for stakeholders. Family caregivers indicated education needs for steps to take in cognitive planning when noticing cognitive changes in a loved one. Healthcare systems (i.e., providers, schedulers, clinical staff) need to make provisions for encouraging family companionship in cognitively impaired older adults’ checkups, offering support and resources for navigation.

